# An Ensemble Prognostic Method of Francis Turbine Units Using Low-Quality Data under Variable Operating Conditions

**DOI:** 10.3390/s22020525

**Published:** 2022-01-11

**Authors:** Ran Duan, Jie Liu, Jianzhong Zhou, Pei Wang, Wei Liu

**Affiliations:** School of Civil and Hydraulic Engineering, Huazhong University of Science and Technology, Wuhan 430074, China; Duan_Ran@hust.edu.cn (R.D.); jie_liu@hust.edu.cn (J.L.); w_sara@hust.edu.cn (P.W.); l_wei@hust.edu.cn (W.L.)

**Keywords:** Francis turbine unit, prognostic, performance state evaluation, degradation trend prediction, DBSCAN, Gaussian mixture model, NSGA-II, Gaussian process regression

## Abstract

The prognostic is the key to the state-based maintenance of Francis turbine units (FTUs), which consists of performance state evaluation and degradation trend prediction. In practical engineering environments, there are three significant difficulties: low data quality, complex variable operation conditions, and prediction model parameter optimization. In order to effectively solve the above three problems, an ensemble prognostic method of FTUs using low-quality data under variable operation conditions is proposed in this study. Firstly, to consider the operation condition parameters, the running data set of the FTU is constructed by the water head, active power, and vibration amplitude of the top cover. Then, to improve the robustness of the proposed model against anomaly data, the density-based spatial clustering of applications with noise (DBSCAN) is introduced to clean outliers and singularities in the raw running data set. Next, considering the randomness of the monitoring data, the healthy state model based on the Gaussian mixture model is constructed, and the negative log-likelihood probability is calculated as the performance degradation indicator (PDI). Furthermore, to predict the trend of PDIs with confidence interval and automatically optimize the prediction model on both accuracy and certainty, the multiobjective prediction model is proposed based on the non-dominated sorting genetic algorithm and Gaussian process regression. Finally, monitoring data from an actual large FTU was used for effectiveness verification. The stability and smoothness of the PDI curve are improved by 3.2 times and 1.9 times, respectively, by DBSCAN compared with 3-sigma. The root-mean-squared error, the prediction interval normalized average, the prediction interval coverage probability, the mean absolute percentage error, and the R2 score of the proposed method achieved 0.223, 0.289, 1.000, 0.641%, and 0.974, respectively. The comparison experiments demonstrate that the proposed method is more robust to low-quality data and has better accuracy, certainty, and reliability for the prognostic of the FTU under complex operating conditions.

## 1. Introduction

With the optimization of global energy structure, hydropower has been vigorously developed [1]. As critical equipment of hydropower utilization, it is important to ensure the safe and stable operation of Francis turbine units (FTUs) [2]. In order to transform the service mode of the FTUs from traditional schedule-based maintenance to state-based maintenance, the prognostic of FTUs has received more and more attention [3]. There are two main parts in the prognostic of FTUs, including performance state evaluation and degradation trend prediction [4]. Based on this, the current and future states of FTUs can be determined so as to formulate a targeted maintenance strategy. However, in practical engineering environments, there are three significant difficulties, including low data quality, complex variable operation conditions, and prediction model parameter optimization.

The state monitoring systems of FTUs work in the harsh environment of high humidity, high vibration, and strong electromagnetic. Due to sensor failure, electromagnetic interference, and missing communication packets, the data quality of the actual measured signal is usually low, which is mainly manifested as data anomaly and data loss [5,6]. Most signal denoising methods, including spectrum analysis and signal reconstruction, are effective for signals with a high sampling rate and consistent sampling frequency [7,8,9]. However, data loss has a great influence on their practical application. Suppression of abnormal data in practical low-quality data sets of FTUs is rarely discussed in existing studies. As an unsupervised learning algorithm, clustering can adaptively detect potential patterns among multi-dimensional data [10]. The density-based spatial clustering of applications with noise (DBSCAN) has the ability to identify the isolated noise from data sets with arbitrary shapes, and the DBSCAN is confirmed to be efficient and robust for low-quality data sets [11,12]. Therefore the DBSCAN is adopted to clean the actual monitoring data set of the FTU in this study.

Generally, FTUs need to participate in the load and frequency regulation of the power grid, and the incoming water levels fluctuate significantly with seasonal variation. Therefore, the operating condition parameters of FTU change frequently [13]. Since the monitoring vibration signals are highly correlated to operation condition parameters, the traditional performance evaluation method with a fixed threshold is difficult to reflect the actual state of FTUs accurately [14]. Machine learning has been widely used in equipment fault diagnosis and performance evaluation due to its good pattern recognition capability [15]. To establish the performance state evaluation model under variable operation conditions, An et al. and Shan et al. adopted the radial basis function and backpropagation neural network (BPNN), respectively, to fit the mapping relationship between water head, active power, and vibration amplitude, as the healthy state model (HSM) of the FTU [4,16]. These studies established the definite functional relation between operation condition parameters and monitoring signal, but the randomness of the signal may still affect the accuracy and stability of these value-to-value mapping models. To deal with it, Rai et al. adopted the Gaussian mixture model (GMM) to fit the probability density distribution (PDD) of vibration features as the HSM of a rolling bearing. The results showed the HSM based on GMM was more accurate and monotonous [17]. However, in this work, the operation condition parameters were constant. Thus, the variable operation condition parameters and the signal randomness are sufficiently considered in this paper, and the GMM is adopted to fit the probability distribution of the running data set as the HSM of the FTU under complex operating conditions.

After constructing the proposed HSM, the real-time state of the FTU can be quantified as performance degradation indicator (PDI) values. Thus, time-series prediction methods can be used to solve the degradation trend prediction problems. For example, Li et al. combined the convolutional filters and the gated recurrent unit to construct a degradation trend prediction model for a turbofan based on data [18]. Jin et al. presented a novel adaptive residual long short-term memory network to predict cutter head torque across domains. The prediction performance is effectively improved by using the knowledge of the source domain dataset [19]. However, most deep learning models only output point prediction values, and it is difficult to quantify the uncertainty of results directly [20]. As a probability prediction model, the Gaussian process regression (GPR) has been widely proven to perform well in uncertain prediction problems [21,22,23]. However, its property is greatly affected by model parameters. Manual parameter tuning is inefficient and relies on prior knowledge. Therefore, some research adopted intelligent optimization algorithms to optimize prediction model parameters automatically [24,25,26]. However, the accuracy of the prediction model was taken as the only optimization objective in these researches. The confidence interval (CI) width represents the certainty of probability prediction, which is also an important objective to consider. As one of the most popular multiobjective optimization algorithms, the non-dominated sorting genetic algorithm (NSGA-II) reduces the complexity of genetic algorithms with fast calculation and good convergence [27,28,29]. Thus the NSGA-II is adopted to optimize the GPR on the two objectives of accuracy and certainty to construct the multiobjective GPR (MOGPR) for degradation trend prediction of FTUs.

According to the above discussion, in the relative field of the prognostic of FTUs, there are few targeted processing methods for low-quality data obtained in practical engineering environments. Meanwhile, existing evaluation methods of FTU under complex conditions rarely consider the randomness of monitoring data. Moreover, in the interval prediction of PDIs, it is still difficult to optimize model parameters automatically while considering both the accuracy and certainty of results.

In this paper, an ensemble prognostic method of FTUs using low-quality data under variable operation conditions is proposed. The major contributions are outlined as follows:

(1) A monitoring data set cleaning approach of FTUs based on DBSCAN is proposed to identify both the singulars and outliers, which enhances the stability and smoothness of the obtained PDI curve.
(2) The running data set of the FTU is constructed by fusing the operation condition parameters and the monitoring data. The HSM is established based on the GMM to realize accurate performance evaluation of the FTU under complex operating conditions to improve the robustness of the performance evaluation model against data missing and data randomness.
(3) Coupling NSGA-II and GPR, the MOGPR is constructed to automatically tune model parameters, avoiding the dependence on prior knowledge, and the accuracy and certainty of probability prediction are improved synchronously.

The remainder of this paper is organized as follows: In Section 2, the proposed approach framework and related theories are expounded. In Section 3, an engineering application of the proposed method is presented. In Section 4, different data cleaning methods, HSM construction, and trend prediction are compared and discussed. Finally, a summary is presented in Section 5.

## 2. Proposed Approach

**Figure 1:**
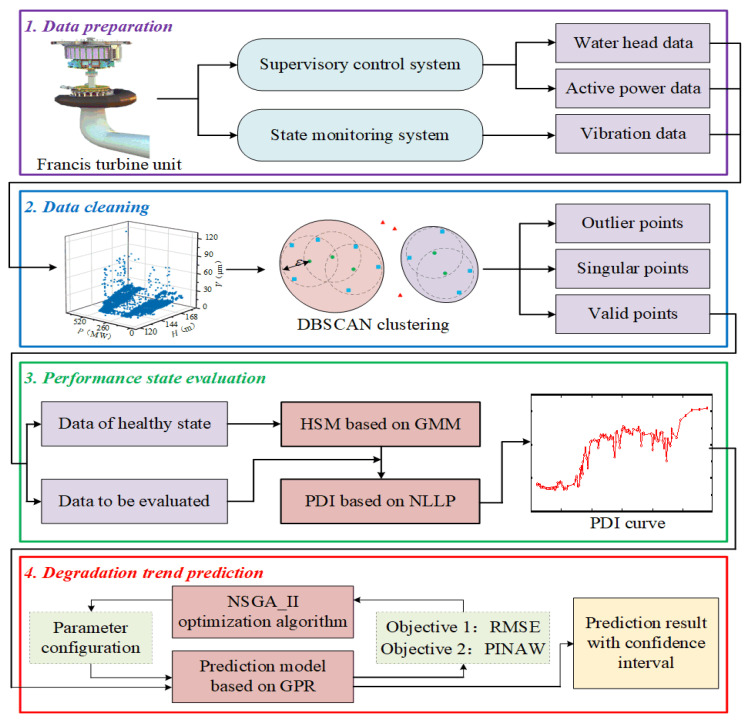
Flowchart of the proposed approach.

To evaluate and predict the performance state of FTUs more accurately under the circumstance of low-quality data and variable operation conditions, an ensemble prognostic method of FTUs using low-quality data under variable operation conditions is proposed in this paper, which mainly includes four steps: data acquisition, data cleaning, performance state evaluation, and degradation trend prediction. The proposed framework flow chart is shown in Figure 1. First, the monitoring data of the water head, active power, and vibration amplitude of the top cover are integrated to construct the running data set of the FTU. Second, aiming at data loss and data anomaly, the data cleaning operation based on DBSCAN is implemented. Third, to solve the problem of monitoring data fluctuating with operation conditions, the HSM is established based on GMM. Then the negative log-likelihood probability (NLLP) is calculated as the PDI of the FTU. The relative trend of PDIs over time reflects the process of performance degradation. Fourth, the MOGPR model is constructed to predict the performance degradation trend of the FTU and takes both prediction accuracy and confidence interval into consideration. Finally, the validity of the performance evaluation model and degradation trend prediction model is evaluated by multi-criterions.

### 2.1. Data Acquisition

Due to their task of regulating the power grid, the operation condition parameters of FTUs change more frequently than other kinds of rotating machines. Therefore, unlike most traditional methods, operation condition parameters are taken into full consideration in the performance state evaluation in this study. The major operation condition parameters of FTUs include rotation speed (*n*), water head (*H*), active power (*P*), flow rate (*Q*), guide vanes opening degree (*α*), etc. Since the duration of transient operation conditions is particularly short compared with the total working time of FTUs, only steady operation conditions are considered in this research. So n can be considered equal to the rated value of the FTU. For a specific FTU, there is a certain relationship between *H*, *P*, *Q*, and *α*. If two of them are identified, others can be inquired from the comprehensive operation curve of the FTU [30]. Thus H and P are chosen as the studied operation condition parameters. As a critical component of the FTU, the top cover is used to seal the runner and support the main shaft. Its vibration amplitude (*V*) can reflect the performance state of the FTU. In conclusion, the running sample set of the FTU is formed by (*H*, *P*, *V*), including both operation condition parameters and monitoring data.

### 2.2. Data Cleaning Based on DBSCAN

**Figure 2:**
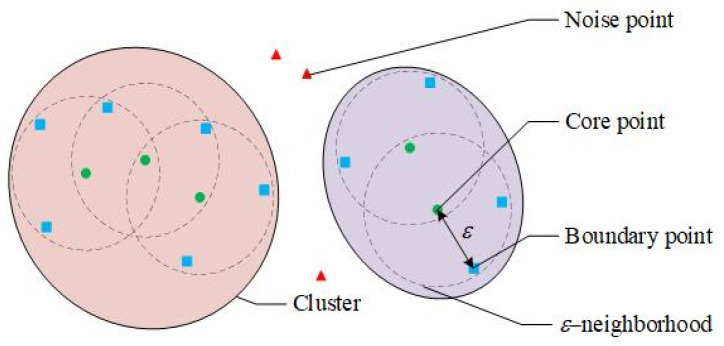
Schematic diagram of the DBSCAN.

To solve the data anomaly in the raw running sample set, a data cleaning approach based on DBSCAN is proposed. Traditional statistics-based methods such as the 3-sigma principle are effective in detecting singular points, but they can hardly identify outliers whose value is within the normal range [31]. DBSCAN clustering algorithm has the advantages of high adaptability, extensible dimension, and applicability to arbitrary data shape [32]. It can automatically identify the singularities and outliers in the multi-dimensional data set. Its schematic diagram is shown in Figure 2, and the main steps are as follows [33]:

**Step 1**: For the sample set $\Phi =\left\{\varphi_{1},\varphi_{2},\cdots ,\varphi_{N}\right\}$, the region whose Euclidean distance from the sample point $\varphi_{i}$ is less than ε is defined as the ε neighborhood of $\varphi_{i}$. If the sample number in the neighborhood of $\varphi_{i}$ is greater than the threshold M, $\varphi_{i}$ is defined as the core point. Samples that are not core points but are in the ε neighborhood of a core point are defined as boundary points. Samples that are not core points and are not boundary points are defined as noise points. The ε neighborhood of a core point is defined as a temporary cluster ${C^{\prime }}_{j}$.

**Step 2**: Traversal the sample set Φ, if the sample point $\varphi_{i}$ in the temporary cluster ${C^{\prime }}_{m}$ is also a core point in another temporary cluster ${C^{\prime }}_{n}$, the union set ${C^{\prime }}_{m}\cup {C^{\prime }}_{n}$ is defined as a new temporary cluster.

**Step 3**: Repeat Step 2 until the sample points in each temporary cluster are all core points or boundary points, then each temporary cluster is determined as a cluster ${C^{\prime }}_{k}$.

### 2.3. Performance State Evaluation Based on GMM

The performance degradation process of the FTU is reflected in the PDD variation of the running sample set (*H*, *P*, *V*). Thus the GMM is adopted to fit the three-dimensional PDD function of the healthy data as the HSM. In GMM, the population distribution of the sample set is assumed to be a combination of a series of Gaussian distributions:

(1) $$P\left(X|\theta \right)=\sum_{k=1}^{K}w_{k}{Ν}_{k}\left(x|\theta_{k}\right)$$

where X represents the healthy data set, $w_{k}$ represents the weight coefficient. $\sum_{k=1}^{K}w_{k}=1$. $\theta_{k}=\left(\mu_{k},\sigma_{k}\right)$ is the distribution parameters. ${Ν}_{k}\left(x|\theta_{k}\right)$ is the PDD function of the *k*^th^ Gaussian component given by:

(2) $${Ν}_{k}\left(x|\theta_{k}\right)=\frac{1}{\sqrt{2\pi }\sigma_{k}}\exp\left(-\frac{{\left(x-\mu_{k}\right)}^{2}}{2\sigma_{k}{}^{2}}\right)$$

The expectation-maximization algorithm is used to estimate $w_{k}$, and $\theta_{k}$ of the GMM [34]:

(3) $$\hat{\theta }=\arg\max P\left(x|\theta \right)=\arg\max\sum_{k=1}^{K}w_{k}{Ν}_{k}\left(x|\theta_{k}\right)$$

where $\hat{\theta }$ is the maximum likelihood estimate value of θ.

The NLLP represents the probability that current data is observed based on the prior given by the GMM. It represents the difference between the PDD of running data $X^{\prime }$ and the constructed HSM. So the NLLP is calculated as the PDI of the FTU.

(4) $$NLLP=-\log P\left(X^{\prime }|\theta \right)$$

### 2.4. Degradation Trend Prediction Based on MOGPR

Through the above procedures, the performance state of the FTU is quantified as PDIs. The performance degradation prediction of the FTU is transformed to a time series prediction task. In this section, the GPR and NSGA-II are combined to construct the MOGPR model for the degradation trend prediction.

#### 2.4.1. GPR Algorithm

As a nonparametric Bayesian inference model, GPR is widely used in probability interval prediction [35,36]. In GPR, the distribution of possible values at each time point is assumed to obey the Gaussian distribution, which can be expressed in terms of the expectation function $\mu_{f}$ and the covariance function κ(·) as:

(5) $$Y=f\left(X\right)∼Ν\left(\mu_{f},\kappa_{ff}\right)$$

where X is the independent variables, κ(·) is also called the kernel function, $\kappa_{ff}=\kappa \left(X,X\right)$.

The joint distribution of the actual observed values $Y^{*}$ and Y also follows the Gaussian distribution.

(6) $$\left[\begin{array}{c} Y\\ Y^{*} \end{array}\right]∼N\left(\left[\begin{array}{c} \mu_{f}\\ \mu_{y} \end{array}\right],\left[\begin{array}{cc} \kappa_{ff} & \kappa_{fy}\\ \kappa_{fy}^{T} & \kappa_{yy} \end{array}\right]\right)$$

where $\kappa_{fy}=\kappa \left(X,X^{*}\right)$, $\kappa_{yy}=\kappa \left(X^{*},X^{*}\right)$.

Finally, the GPR model can be expressed as:

(7) $$Y∼N\left(\kappa_{fy}^{T}\kappa_{ff}^{-1}Y^{*}+\mu_{f},\kappa_{yy}-\kappa_{fy}^{T}\kappa_{ff}^{-1}\kappa_{fy}\right)$$

The kernel functions κ(·) are used to enhance the representation of relationships between input samples. Various kernel functions have different properties and characteristics. The commonly used kernel functions include radial basis function (RBF), matern (MA), rational quadratic (RQ). The kernel function adopted in this study is composed of them.

(8) $$\kappa_{RBF}\left(X_{i},X_{j}\right)=\exp\left(-\frac{{\left\|X_{i}-X_{j}\right\|}^{2}}{2l^{2}}\right)$$

(9) $$\left\{ \begin{array}{l} \kappa_{MA}\left(X_{i},X_{j}\right)=\frac{1}{\sqrt{2\pi }}\rho^{1.5}\left(1+\frac{\sqrt{3}\rho }{l}\right)\exp\left(-\frac{\sqrt{3}\rho }{l}\right)\\ \rho =\frac{\sqrt{3}}{l}\left\|X_{i}-X_{j}\right\| \end{array}\right.$$

(10) $$\kappa_{RQ}\left(X_{i},X_{j}\right)=1+\frac{{\left\|X_{i}-X_{j}\right\|}^{2}}{2l^{2}}$$

These kernel functions all contain a length scale l. To improve the performance of the model, the common approach is to adjust l manually, which is less efficient. Aiming at this problem, the NSGA-II algorithm is introduced to automatically optimize the GPR model. Since GPR outputs the PDD of predicted values, quantiles and confidence intervals (CIs) can be calculated directly. Interval prediction results are usually evaluated by multiple criteria, including the root-mean-square error (RMSE), which reflects the accuracy, and the prediction interval normalized average (PINAW), which reflects certainty, defined as:

(11) $$RMSE=\sqrt{\frac{1}{N}\sum_{i=1}^{N}{\left({\hat{y}}_{i}-y_{i}{}^{*}\right)}^{2}}$$

(12) $$PINAW=\frac{1}{N\Delta y^{*}}\sum_{i=1}^{N}\left(u_{i}-l_{i}\right)$$

where N is the number of series data, $\hat{y}$ and $y^{*}$ represents the prediction value and the actual value, respectively. $\Delta y^{*}$ is the difference between the maximum and minimum values of the actual series, $u_{i}$ and $l_{i}$ represents the upper and lower boundary of 95% CI. Lower RMSEs and PINAWs indicate better accuracy and certainty of the prediction model.

#### 2.4.2. NSGA-II Algorithm

**Figure 3:**
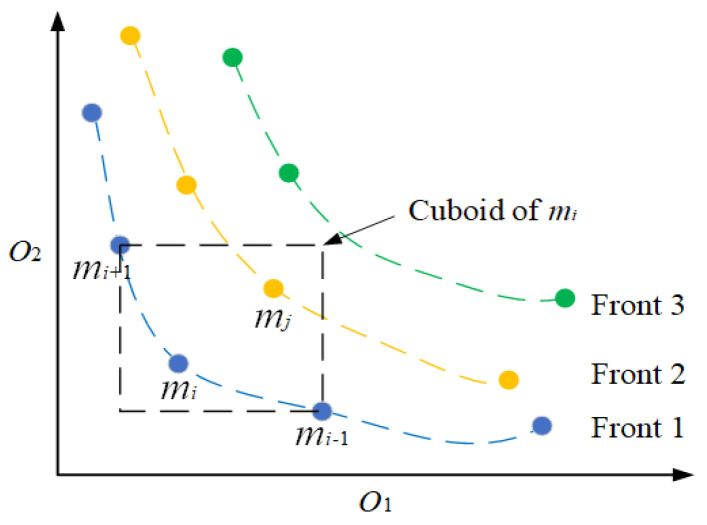
Non-dominated sorting and crowding distance.

NSGA-II makes excellent improvements on NSGA, and it has faster computational efficiency and population diversity [37]. There are two basic concepts in NSGA-II: non-dominated sorting and crowding distance. The procedure of non-dominated sorting begins with the identification of non-dominated solutions. As shown in Figure 3, for two members $m_{i}$ and $m_{j}$, if all the objectives of $m_{i}$ are better than $m_{j}$, $m_{i}$ is defined to dominate $m_{j}$. Then, the members which are not dominated by others constitute the current front. Next, the members of the current front are removed, and the sorting is performed on the remained population. The procedure is repeated until all the members are distributed to different fronts.

The crowding distance is used to measure the density of members. It is defined as the sum of the side length of the cuboid shown in Figure 3.

(13) $$cd\left(m_{i}\right)=\sum_{k=1}^{K}\left|o_{k}^{i+1}-o_{k}^{i-1}\right|$$

where *K* is the dimension number of the objectives. Selecting members with a high crowding distance can improve the diversity of the population.

**Figure 4:**
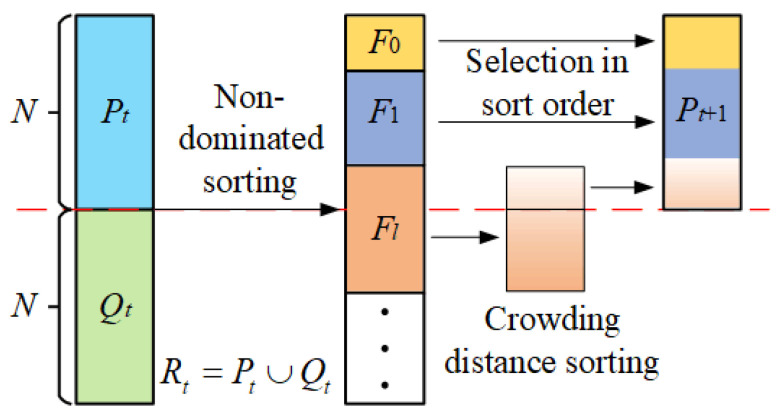
Schematic diagram of the NSGA-II.

The brief schematic of NSGA-II is illustrated in Figure 4, and the main steps are as follows:

**Step 1**: The population is initialized. Then, the offspring population *Q_t_* is generated from the parental population *P_t_* through crossover and mutation operations. The population sizes of *P_t_* and *Q_t_* are both *N*.

**Step 2**: The *P_t_* and *Q_t_* are merged to form the *R_t_*. The non-dominated sorting is performed on *R_t_*, and a series of fronts *F_i_* are obtained.

**Step 3**: The fronts are selected in sort order to form the *P_t_*_+1_ until the population size of *P_t_*_+1_ exceeds *N*. The members of the last front *F_l_* are sorted by the crowding distance. The members are selected in order until the population size of *P_t_*_+1_ equals *N*.

**Step 4**: The above steps are repeated until the maximum number of evolution is reached. Then the first front is selected as the Pareto front.

#### 2.4.3. MOGPR Model

**Figure 5:**
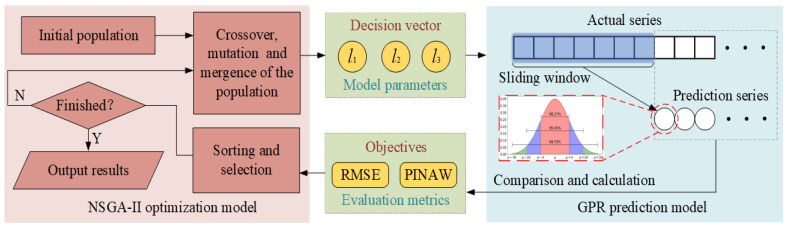
Schematic diagram of the MOGPR.

The GPR and the NSGA-II are coupled to construct the MOGPR model. The three-dimensional decision vector is formed by the length scales of RBF, MA, and RQ in the GPR model. The multiple objectives include both RMSE and PINAW. The schematic diagram of the MOGPR is illustrated in Figure 5. The GPR model sets parameter values according to the decision vector generated by NSGA-II and calculates the prediction results. Then the results are compared with the actual values to obtain multi objectives. NSGA-II updates population location according to the multi objectives. Through several epochs of evolution, the Pareto front of the optimal result is finally output.

## 3. Engineering Application

In this section, the long-term monitoring data of a large actual FTU is adopted to conduct the experiments. The basic information of the FTU and the data source are described first. Then the data cleaning based on DBSCAN is implemented on the raw running data set. Next, the HSM of the FTU under variable operation conditions is constructed based on GMM. Finally, the performance degradation trend of the FTU is predicted by the proposed MOGPR.

### 3.1. Data Description

**Figure 6:**
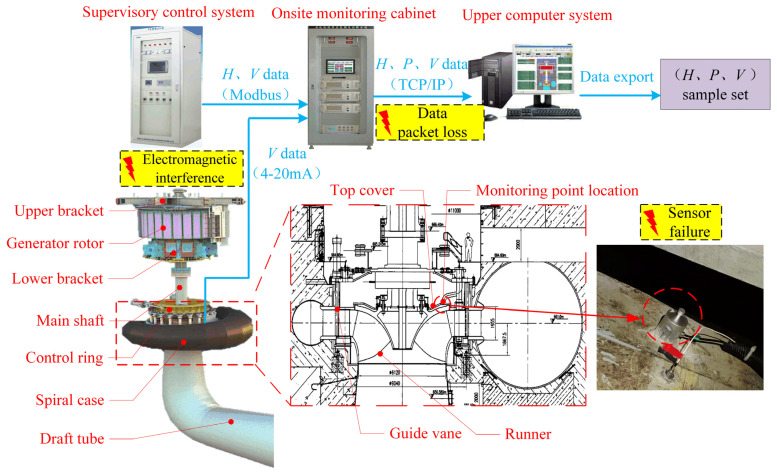
Structure of the FTU and the data acquisition system.

The studied FTU is located in the Dadu River basin, Sichuan, China. Its basic parameters are listed in Table 1. A set of PSTA-2100 state monitoring systems, including on-site monitoring cabinet and upper computer system, is configured on the FTU. The on-site monitoring cabinet is formed by a sensor power module, data acquisition module, synchronous clock module, and industry cabinet. It is located near the FTU. The top cover vibration (*V*) is monitored by the acceleration sensor (PCB 352A60). The water head (*H*) and the active power (*P*) are obtained through communication with the supervisory control system of the plant following the modbus 485 protocol. The collected data are transmitted to the upper computer through the communication link following TCP/IP protocol. The actual length of the communication link, which consists of optical cables, switches, routers, repeaters, and transceivers, is above 1000 m. The analyzed sample set (*H*, *P*, *V*) is constructed by the data records exported from the upper computer. The overall structure of the FTU and the data acquisition system are illustrated in Figure 6. To sum up, the data acquisition process involves a series of devices and processes, which are prone to data loss and data abnormality.

**Figure 7:**
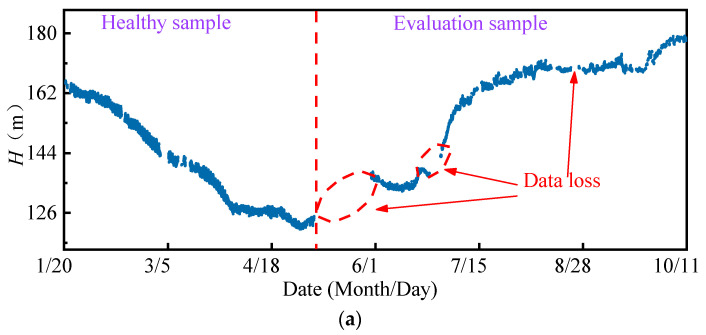

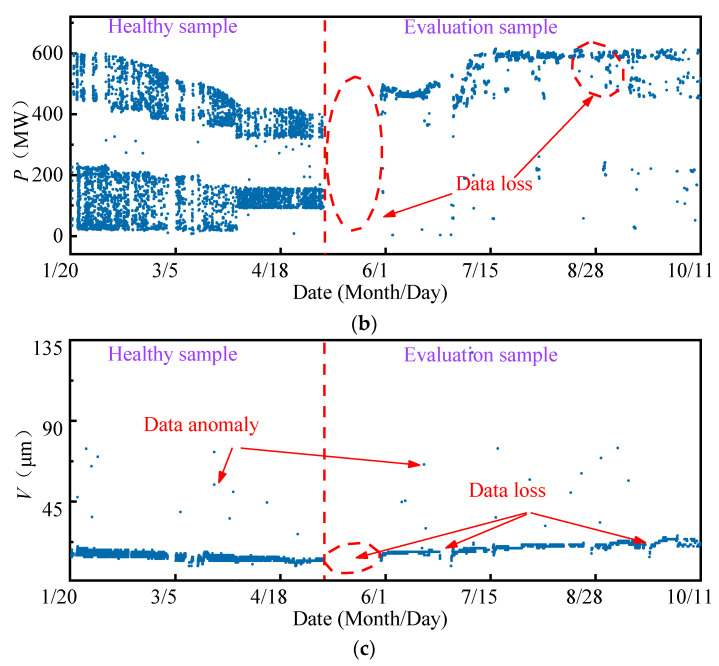
On-site measured data: (**a**) Water head; (**b**) Active power; (**c**) Vibration amplitude of top cover.

**Figure 8:**
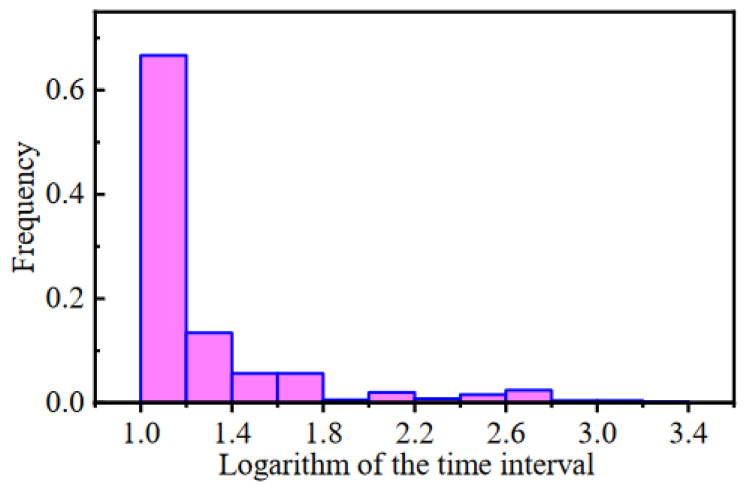
Frequency distribution histogram of logarithms of time intervals.

The exported data includes 104,454 historical records from 20 January 2019 to 11 October 2019 for a total of 264 days, as shown in Figure 7. The on-site dataset is quite different from the high-quality data obtained in the ideal experimental environment, such as the bearing life cycle dataset published by the Center for Intelligent Maintenance Systems, University of Cincinnati [38]. Most experimental data sets are less disturbed by external interference and have stable sampling frequency. However, it can be seen that the data loss and data anomaly are very obvious in the raw on-site data set. Data loss leads to unequal time stamps of samples. As shown in Figure 8, the time intervals between adjacent samples ranged from 10 s to 10^3^ s, which makes it difficult to analyze the frequency domain features of signals. In addition, because of the task of grid regulation and seasonal changes in the water head, the operation condition parameters of the FTU change frequently and drastically. Low data quality and variable operation conditions greatly influence the performance state evaluation and degradation trend prediction of the FTU.

### 3.2. Data Cleaning

**Figure 9:**
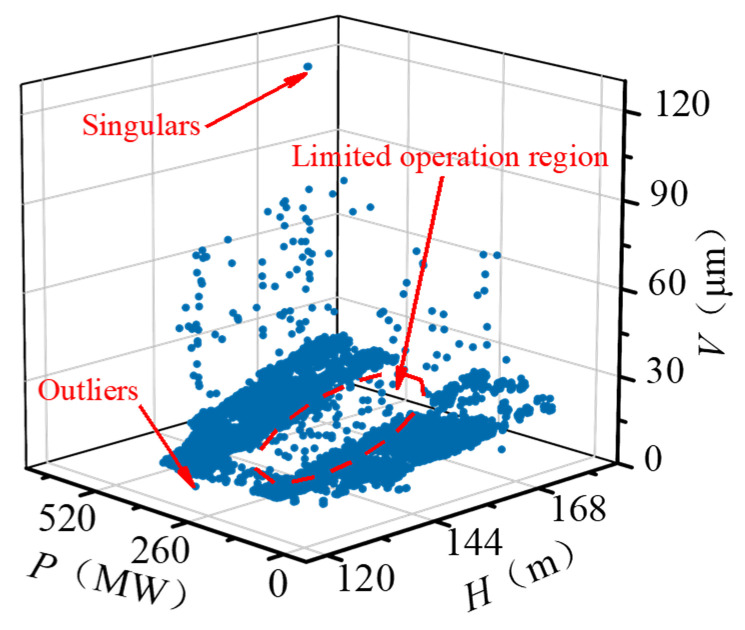
Raw running sample set.

**Figure 10:**
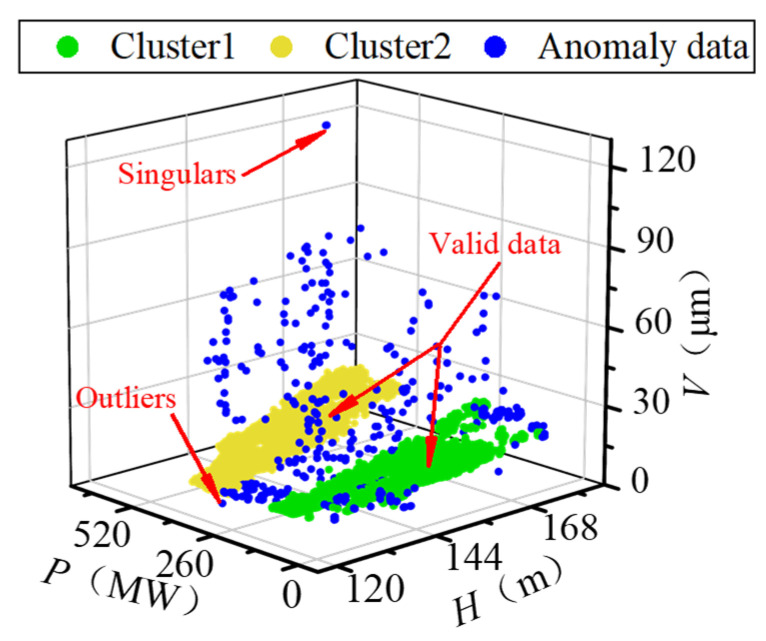
Processing result of DBSCAN.

The raw running sample set (*H*, *P*, *V*) is exhibited in Figure 9. Due to the characteristic of the FTU, there is a specific limited operation region within the operating conditions. In the limited area, the stability and efficiency of the FTU decrease. In practice, the FTU is avoided from working in the limited area, so the sample points are concentrated in two regions. Because of various interference factors, the data anomaly is very obvious in the raw data set. The data anomaly mainly includes singular points whose values deviate significantly from the normal level and outliers whose values are within the normal scope, but their distribution deviates from the valid samples. To reduce the impact of data anomaly, DBSCAN was adopted to identify the singulars and outliers in the original sample set. The ε and *M* were set as 7.5 and 200, respectively. The processing result is shown in Figure 10. The two sample concentrated regions are marked as cluster 1 and cluster 2. Singulars and outliers are both marked as anomaly data automatically. Since the DBSCAN considers the distribution density of samples rather than the value of data, it performs well in identifying both outliers and singularities in the low-quality data set.

### 3.3. Performance State Evaluation of the FTU

Routine maintenance was implemented before 20 January 2019 on the studied FTU, and the restart test run performed normally. Moreover, as shown in Figure 7, the period from 20 January to 6 May 2019 includes most of the possible operating conditions of the FTU. Therefore, a total of 58,175 valid data points during this period were selected to construct the healthy sample set.

**Figure 11:**
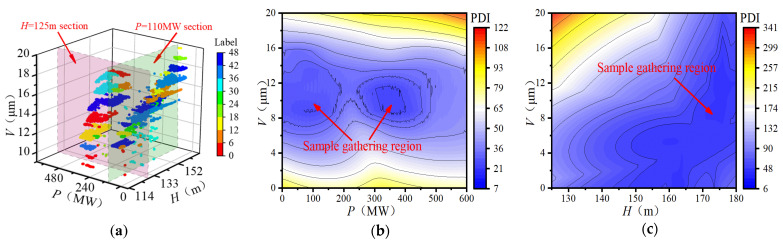
HSM based on GMM (**a**) Result of GMM clustering (**b**) PDI distribution on *H* = 125 m section; (**c**) PDI distribution on *P* = 110 MW section.

The GMM was adopted to fit the three-dimensional PDD function of the healthy sample set for the HSM construction of the FTU, as illustrated in Figure 11a, where different colors represent different Gaussian components of the HSM. On this basis, the NLLP was calculated as the PDI of the FTU. The distribution of PDIs on the *H* = 125 m section and *P* = 110 MW section of the HSM is shown in Figure 11b,c. It shows that there is a complex mapping relationship between PDIs and operation condition parameters. The PDIs are relatively lower in regions with dense distribution of healthy samples.

**Figure 12:**
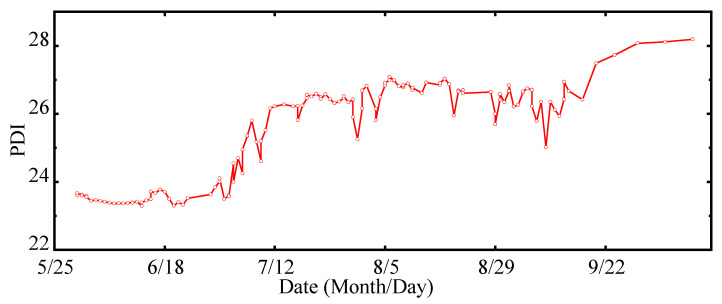
Performance degradation curve of the FTU.

The valid data after 6 May 2019 were selected to construct the evaluation sample set. All 300 evaluation samples were input into the HSM as a group to calculate the PDIs. The timestamp corresponding to the last evaluation sample in each group was taken as the evaluation time. The obtained performance degradation curve, including 135 PDIs, is demonstrated in Figure 12. Although the PDI curve fluctuates locally due to low data quality, the curve has an obvious upward trend reflecting the performance degradation process of the FTU.

### 3.4. Degradation Trend Prediction of the FTU

To forecast the performance degradation trend of the FTU, a rolling prediction model based on MOGPR was established. Every seven PDIs, as the time window, were input into the prediction model, and the next PDI was predicted. The sliding step was set as one PDI point. As the time window slides, the prediction model is constantly updated.

**Figure 13:**
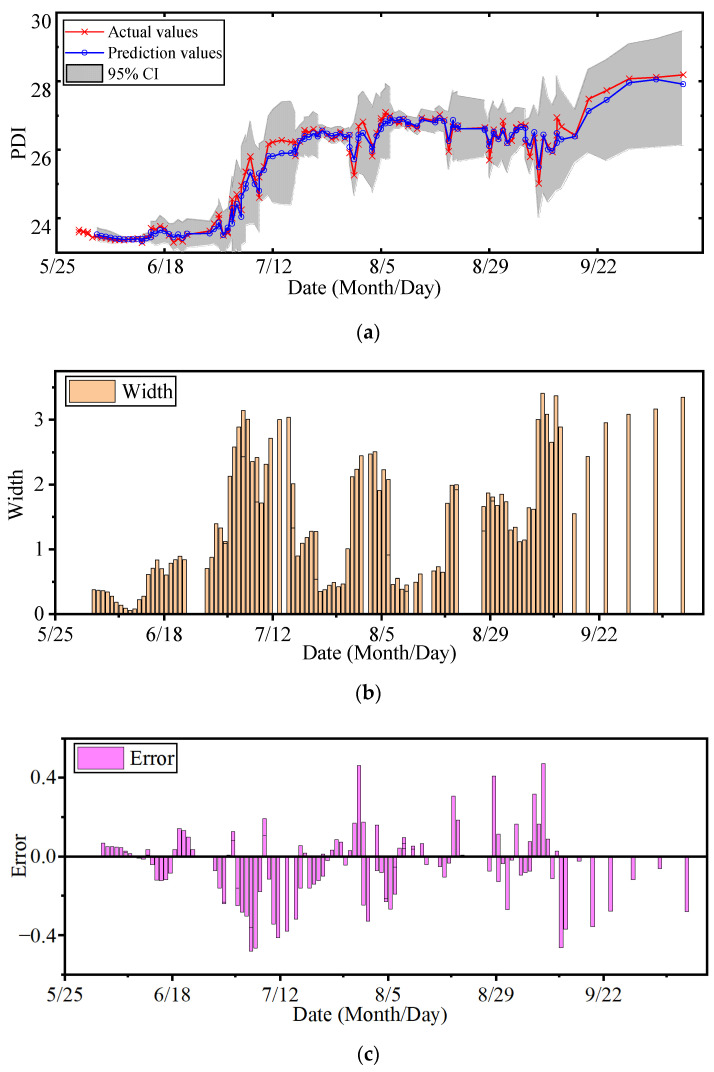
Prediction result of MOGPR: (**a**) Prediction result; (**b**) Width of CI; (**c**) Error distribution.

The RBF, MA, and RQ were chosen to construct the kernel function of the GPR model. The length scales $l_{RBF}$, $l_{MA}$ and $l_{RQ}$ were optimized with the NSGA-II according to two objectives of RMSE and PINAW. The search scopes of the above three length scales were all set as [10^−5^, 1]. The parameter configuration of the NSGA-II is listed in Table 2. The prediction result that is closest to the origin in the Pareto front of multiobjective optimization is exhibited in Figure 13. The proposed MOGPR takes both accuracy and certainty of interval prediction into account. The maximum error does not exceed 0.47, and the maximum width of CI does not exceed 3.41.

## 4. Comparison Analyses

To validate the effectiveness of the proposed approach, different methods of data cleaning, HSM construction, and trend prediction were compared. The experiments were conducted by Python 3.7.9 in a calculation station with an Intel Core i9-10900K CPU, an NVIDIA GeForce RTX 2080 Super GPU, and 64 GB RAM.

### 4.1. Comparison of Different Data Cleaning Methods

**Figure 14:**
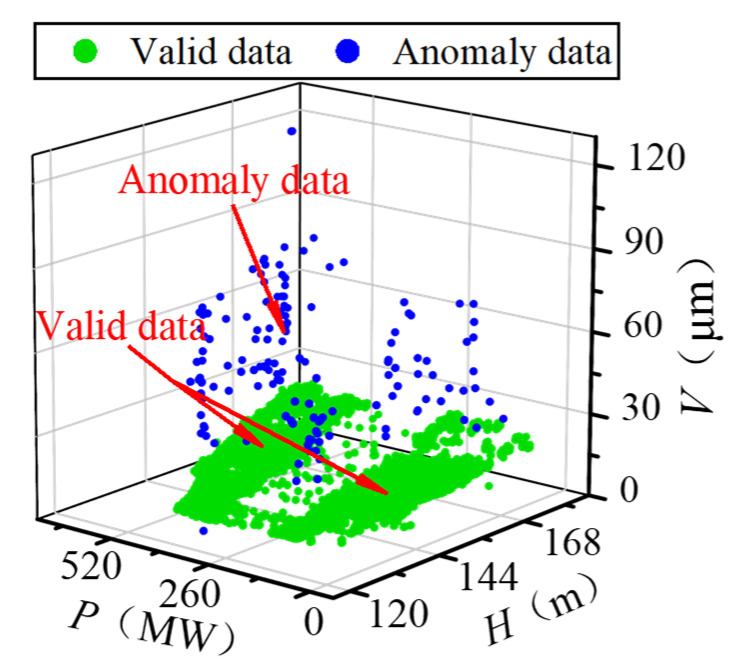
Processing result of 3-sigma principle.

To validate the effectiveness of the proposed data cleaning method, the DBSCAN and 3-sigma principle are compared in this section. Different data sets were adopted to establish HSMs, including the raw data set $\Phi_{0}$, the 3-sigma-processed data set $\Phi_{1}$, and the DBSCAN-processed data set $\Phi_{2}$. The processing result of the 3-sigma principle is shown in Figure 14. Compared with Figure 10, it can be discovered that the 3-sigma principle can recognize partial singularities but can hardly identify outliers.

Then the PDI curves were calculated in the same way as Section 3.3. The *STD* and *S* were adopted as the criteria of stability and smoothness, defined as follows:

(14) $$STD=\sqrt{\frac{1}{N}\sum_{i=1}^{N}{\left(I_{i}-\bar{I}\right)}^{2}}$$

(15) $$S=\frac{\sum_{i=1}^{N-1}\left|I_{i+1}-I_{i}\right|}{N-1}$$

where *I* represents the PDI and *N* is the number of PDIs in the curve. Smaller *S* and *STD* indicate that the curve is smoother and the represented performance degradation trend is clearer.

**Figure 15:**
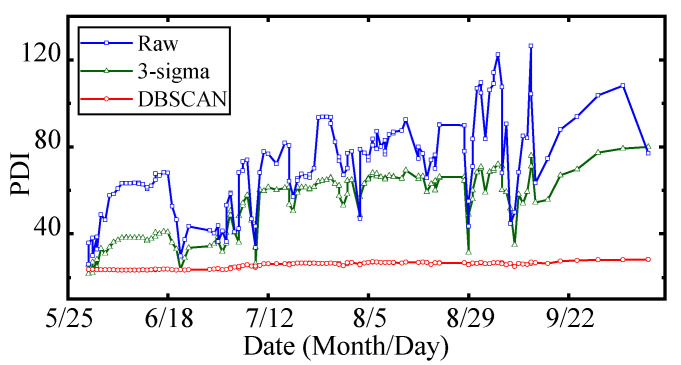
Comparison of different data cleaning methods.

The comparison results are shown in Table 3 and Figure 15. It can be seen that:

(1) Due to the influence of data anomaly, the performance degradation curve obtained by the raw data set presents many mutation points, which is difficult to accurately reflect the real performance state of the FTU.
(2) There are 28.7% and 50.2% decreases in the *STD* and *S* of the raw PDI curve, respectively, using 3-sigma, while there are 93.1% and 97.2% decreases in the *STD* and *S,* respectively, using DBSCAN. The stability and smoothness of PDI curves are improved by 3.2 times and 1.9 times, respectively, by DBSCAN compared with 3-sigma
(3) After data cleaning based on DBSCAN, the calculated PDI curve has the lowest *STD* and *S* (1.42 and 0.24), which indicates the curve reveals the performance degradation trend more clearly.

### 4.2. Comparison of Different Methods for HSM Construction

To compare the robustness of HSMs towards data loss, different ratios (*r*) of randomly selected healthy samples were adopted to build HSMs based on GMM, SVM, and BPNN, respectively, r∈[0.2,0.4,0.6,0.8,1]. The major parameters of the models mentioned above are listed in Table 4. Then the entire healthy sample set was input into the HSMs to calculate PDIs. Each experiment was repeated 100 times, and the STDs of results were calculated. In order to make the PDI results of different HSMs comparable, the maximum-minimum normalization was implemented to transform the value range of PDIs to [0, 1]. Unlike GMM, SVM and BPNN establish the mapping relationship between operation condition parameters and vibration amplitude values. Thus the PDI of HSMs based on SVM and BPNN was defined as:

(16) $$PDI=\frac{1}{N}\sum_{i=1}^{N}\left|V^{*}-\hat{V}\left(H^{*},P^{*}\right)\right|$$

where $V^{*}$, $H^{*}$, and $P^{*}$ represent the real values of vibration amplitude, water head, and active power. $\hat{V}$ denotes the mapping value outputted by the HSM.

**Figure 16:**
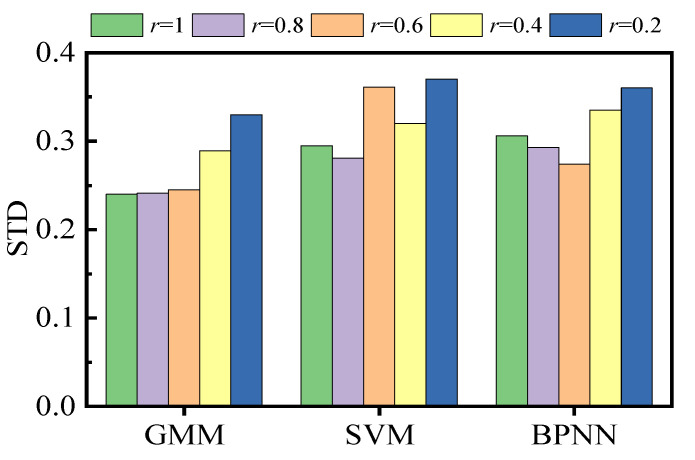
Comparison of different HSM.

The comparison results are illustrated in Figure 16. As the sample quantity decreases, the results’ STD based on GMM increases slowly when *r* is greater than 0.6. The results’ STDs based on SVM and BPNN are relatively higher and have no significant relationship with the data quantity of HSM construction. Since the HSM based on GMM looks at the PDD of samples rather than the specific values of individual samples, the actual distribution of the population can be estimated from the sample distribution as long as the total quantity of samples is enough. Therefore, the proposed HSM is less affected by data loss.

### 4.3. Comparison of Different Prediction Methods

To verify the effectiveness of the proposed MOGPR, different prediction models are compared, including two multiobjective optimization machine learning models named MOQRLSTM and MOQRNN, two machine learning models without parameter optimization named QRLSTM and QRNN [39], three nonparametric statistical regression models named locally weighted linear regression (LWLR) [40] and kernel regression (KR) [41]. The loss functions of QRLSTM and QRNN are defined as quantile regression functions [42], which makes them predict conditional quantiles. According to the two objectives of RMSE and PINAW, the node numbers of each hidden layer of QRLSTM and QRNN are optimized by NSGA-II to construct the MOQRLSTM and MOQRNN. The NSGA-II is set as the same as Section 3.4. LWLR and KR are widely used to predict time series data with trend terms of different scales because their algorithms are simple, and the time costs are low. The main parameters of the mentioned models are listed in Table 5.

For a more complete performance comparison of the various models, in addition to the two objectives of RMSE and PINAW, the other fourcriteria named prediction interval coverage probability (PICP), mean absolute percentage error (MAPE), R2_score, and probability integral transform (PIT) are introduced [43]. The RMSE represents the accuracy of prediction results; the PINAW indicates the certainty of the prediction interval. Their definitions are given in Equations (11) and (12), respectively. The MAPE is complementary to RMSE and reflects the relative accuracy of the prediction, defined as:

(17) $$MAPE=\frac{1}{N}\sum_{i=1}^{N}\left|\frac{{\hat{y}}_{i}-y_{i}{}^{*}}{y_{i}{}^{*}}\right|\times 100\%$$

The R2_score represents the proportion that can be explained by the fitted regression relationship in the overall change trend of the dependent variable, defined as:

(18) $$R2\_score=\frac{{\sum_{i=1}^{N}\left({\hat{y}}_{i}-\bar{y}\right)}^{2}}{{\sum_{i=1}^{N}\left(y^{*}{}_{i}-\bar{y}\right)}^{2}}$$

where $\bar{y}$ is the mean of the actual value $y^{*}$. A R2_score close to 1 indicates that the prediction model is more reliable.

The PICP represents the probability that the predicted CI contains the observed values, expressed as:

(19) $$\begin{array}{c} PICP=\frac{1}{N}\sum_{i=1}^{N}f_{i}\\ f_{i}=\left\{ \begin{array}{l} 0,y_{i}^{*}\notin [l_{i},u_{i}]\\ 1,y_{i}^{*}\in [l_{i},u_{i}] \end{array}\right. \end{array}$$

where $f_{i}$ indicates whether the observed value is within the predicted CI. A higher PICP indicates the interval prediction result is more accurate.

The PIT values reflect the correlation between the prediction distribution and the actual distribution, defined as:

(20) $$PIT=\int_{-\infty }^{y^{*}}\hat{P}\left(y\right)\;dy$$

where $\hat{P}$ is the prediction distribution and $y^{*}$ is the observed value. If the distribution of PIT values is similar to the uniform distribution, the prediction reliability is better.

**Figure 17:**
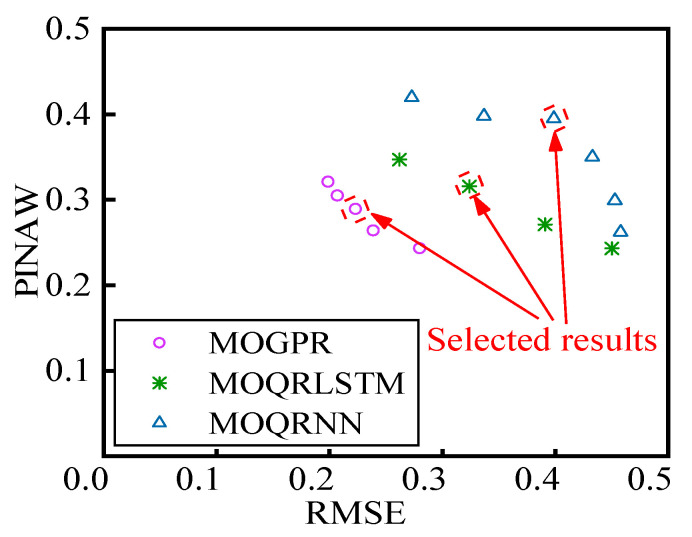
Pareto fronts of optimization results.

The Pareto fronts of optimization results of MOGPR, MOQRLSTM, and MOQRNN are illustrated in Figure 17. The results of MOGPR are generally closer to the origin. The lowest PINAW values reached by the 3 models are similar (about 0.243~0.271), while the lowest RMSE value obtained by MOGPR (about 0.199) is smaller than MOQRLSTM’s (about 0.262) and MOQRNN’s (about 0.273). As two independent optimization objectives, RMSE and PINAW have the same dimension of quantity and similar value range. In this study, the importance of the two objectives is considered equal. So the result closest to the origin in the Pareto front is selected as the final result of three multiobjective optimization prediction models.

**Figure 18:**
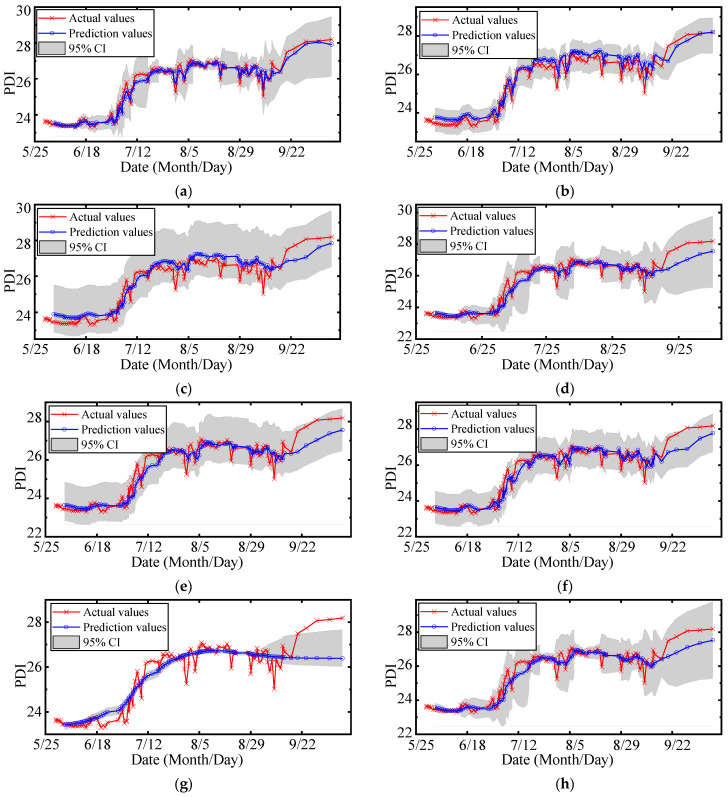
Results of interval prediction: (**a**) MOGPR; (**b**) MOQRLSTM; (**c**) MOQRNN; (**d**) GPR; (**e**) QRLSTM; (**f**) QRNN; (**g**) LWLR; (**h**) KR.

**Figure 19:**
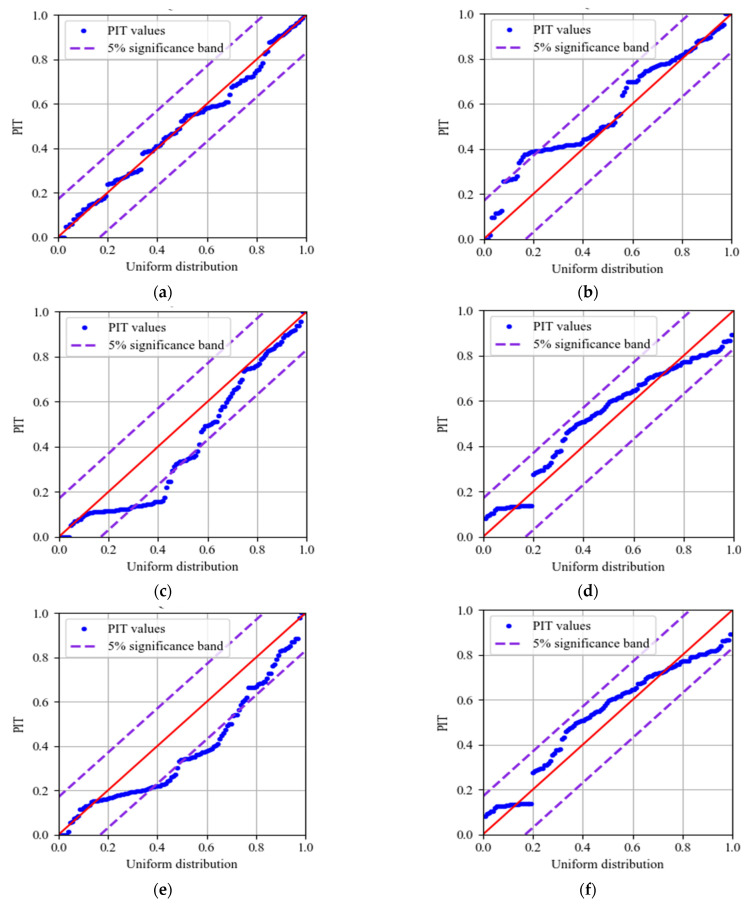

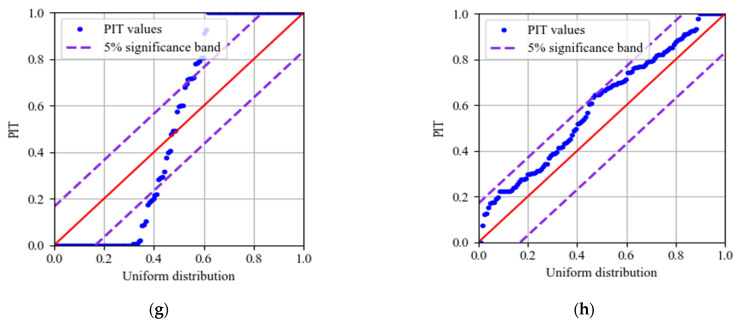
QQ plot of PITs: (**a**) MOGPR; (**b**) MOQRLSTM; (**c**) MOQRNN; (**d**) GPR; (**e**) QRLSTM; (**f**) QRNN; (**g**) LWLR; (**h**) KR.

The multi metrics of different models are listed in Table 6, including RMSE, PINAW, PICP, MAPE, R2_score, and cost time. The prediction results are shown in Figure 18, and the quantile-quantile (QQ) plots of PITs are illustrated in Figure 19. The areas between the purple dotted lines in QQ plots represent PITs that follow the uniform distribution at a 5% significance level. The analysis of the results is as follows:

(1) The RMSE and the MAPE of MOGPR are the lowest (0.223 and 0.641%), and the R2_score of MOGPR is the highest (0.974). As shown in Figure 18a, MOGPR has a good prediction effect on both long-term trends and local fluctuation of the PDI curve. It proves that the prediction accuracy of MOGPR is the best.
(2) The PINAW of LWLR is the lowest (0.080), but the RMSE of LWLR is the highest (0.472). As can be seen from Figure 18g, LWLR cannot effectively forecast the local fluctuation of data. Except for LWLR, the PINAW of MOGPR is lower than other models, which demonstrates that MOGPR has better certainty.
(3) The RMSEs, PINAWs, MAPEs, and R2_scores of multiobjective optimization models (MOGPR, MOQRLSTM, and MOQRNN) are improved compared with original models (GPR, QRLSTM, and QRNN), which indicates that the multiobjective optimization process can effectively improve the performance of the original models.
(4) The PICPs of most models are higher than 0.95, apart from LWLR. The PICPs of MOGPR, GPR, and KR achieve 1.0, which means all the actual points fall within the prediction 95% CIs. It also indicates that compared with other criteria, PICP is easier to reach the maximum value, so it is not suitable to be an optimization objective.
(5) Because of the process of parameter optimization, the cost time of the three multiobjective optimization prediction models (MOGPR, MOQRLSTM, and MOQRNN) is obviously higher. Among these three models, MOGPR has the fastest calculation speed. Because compared with neural network algorithms, the GPR has fewer parameters.
(6) As shown in Figure 19, the PIT values’ distribution of MOGPR is closest to the uniform distribution, which is displayed as the red diagonal in the QQ plot. In addition, all PIT points are located within the 5% significance band, which indicates that the prediction CI of MOGPR is reliable. The PITs of the LWLR distribute around 0 or 1, which indicates that the LWLR is most unsuitable for interval prediction of PDI compared with other models.

In summary, among the eight compared models, MOGPR has better accuracy, certainty, and reliability of probability prediction. Besides, the calculation effectiveness of MOGPR is higher than other multiobjective optimization prediction models.

## 5. Conclusions

In this study, aiming at the three major practical engineering difficulties of low data quality, complex variable operation conditions, and prediction model parameter optimization, an ensemble prognostic method of FTUs using low-quality data under variable operation conditions is proposed. Firstly, to comprehensively reflect the performance of the FTU under complex operation conditions, the running data set is constructed by combining operation condition parameters and monitoring data. Secondly, to reduce the impact of anomaly data, the DBSCAN is adopted to clean both outliers and singulars in the raw running data set. Thirdly, based on the GMM and the probability theory, the HSM is established, which improves the robustness of the evaluation model against data missing and data randomness. Fourthly, the MOGPR is proposed to predict the performance degradation trend with a confidence interval and to automatically optimize model parameters on both accuracy and certainty. Finally, a series of comparison experiments were implemented on practical data set from an actual large FTU.

The experimental results demonstrate that: (1) The data cleaning approach based on DBSCAN performs better in identifying both outliers and singularities. The stability and smoothness of PDI curves are improved by 3.2 times and 1.9 times, respectively, by DBSCAN compared with 3-sigma. (2) Compared with SVM and BPNN, the HSM based on GMM has better robustness against data loss. (3) The proposed MOGPR has better accuracy, certainty, and reliability of probability prediction. The root-mean-squared error, the prediction interval normalized average, the prediction interval coverage probability, the mean absolute percentage error, and the R2 score of the proposed method achieved 0.223, 0.289, 1.000, 0.641%, and 0.974, respectively.

Thus, the proposed method can be applied to the performance evaluation and degradation trend prediction of FTUs in a practical engineering environment. In further research, if the running data across multiple maintenance periods are available, the corresponding relationship between PDI values and actual state can be established according to maintenance records. On this basis, the multi-level degradation state of the FTU can be divided, and corresponding maintenance strategies can be formulated to provide technical support for state-based maintenance.

## Figures and Tables

**Table 1:** Basic parameters of the FTU.

Parameters	Values	Units
Inlet diameter of the runner	6964	mm
Number of runner blades	15	\
Number of guide vanes	24	\
Rated power	611	MW
Rated rotate speed	125	r/min
Rated flow rate	435	m^3^/s
Rated water head	156.7	m

**Table 2:** Parameter configuration of the NSGA-II.

Parameter	Value
Population size	10
Max number of evolutions	20
Crossover rate	0.8
Mutation rate	0.2

**Table 3:** Comparison of different data cleaning methods.

Data Set	Method	*STD*	*S*
*Φ*_0_	Raw data	20.61	8.65
*Φ*_1_	3-sigma principle	14.70	4.31
*Φ*_2_	DBSCAN	1.42	0.24

**Table 4:** Parameter setting of compared models.

Model	Parameter	Value
GMM	Component number	50
SVM	Kernel type	RBF
	Regularization parameter	1.0
	Epsilon	0.2
BPNN	Number of layers	3
	Number of layer nodes	20
	Learning rate	0.01
	Epochs	100

**Table 5:** Parameter setting of compared models.

Model	Parameter	Range or Value ^1^
MOGPR	Kernel length scale	[10^−5^, 1]
	Kernel type	RBF+MA+RQ
MOQRLSTM	Number of layer nodes	[5, 60]
	Number of layers	3
MOQRNN	Number of layer nodes	[5, 60]
	Number of layers	3
GPR	Kernel length scale	10^−4^
	Kernel type	RBF+MA+RQ
QRLSTM	Number of layer nodes	20
	Number of layers	3
QRNN	Number of layer nodes	20
	Number of layers	3
LWLR	Estimating fraction	0.4
KR	Kernel type	Gaussian kernel

^1^ For the three multiobjective optimization prediction models, some parameters are optimized in a range. For the five prediction models without parameter optimization, the parameters are certain values.

**Table 6:** Results of comparison experiments.

Model.	RMSE	PINAW	PICP	MAPE (%)	R2_Score	Time (s)
MOGPR	**0.223**	0.289	**1.000**	**0.641**	**0.974**	1086.1
MOQRLSTM	0.324	0.316	0.957	1.001	0.946	19,408.1
MOQRNN	0.399	0.395	0.961	1.252	0.918	18,639.2
QRLSTM	0.408	0.448	0.984	1.148	0.914	137.2
QRNN	0.417	0.394	0.953	1.143	0.910	114.3
GPR	0.378	0.373	**1.000**	1.064	0.926	2.3
LWLR	0.472	**0.080**	0.348	1.231	0.885	1.4
KR	0.378	0.373	**1.000**	1.025	0.926	6.2

## Data Availability

Not applicable.

## Abbreviations

FTU	Francis turbine unit
NLLP	Negative log-likelihood probability
DBSCAN	Density-based spatial clustering of applications with noise
BPNN	Backpropagation neural network
HSM	Healthy state model
GMM	Gaussian mixture model
PDD	Probability density distribution
PDI	Performance degradation indicator
GPR	Gaussian process regression
MOGPR	Multi-objective GPR
CIs	Confidence intervals
NSGA-II	Non-dominated sorting genetic algorithm
RMSE	Root-mean-square error
PINAW	Prediction interval normalized average
LWLR	Locally weighted linear regression
KR	Kernel regression
